# Therapeutic Options of Chondrodermatitis Nodularis Helicis

**DOI:** 10.1155/2016/4340168

**Published:** 2016-01-27

**Authors:** Lea Juul Nielsen, Caroline Holkmann Olsen, Jørgen Lock-Andersen

**Affiliations:** ^1^Department of Plastic Surgery and Breast Surgery, Roskilde Hospital, 4000 Roskilde, Denmark; ^2^Department of Pathology, Roskilde Sygehus, Denmark

## Abstract

Chondrodermatitis Nodularis Helicis is a benign inflammatory process affecting the skin and cartilage of the ear. It typically presents as a painful nodule surrounded by an area of erythema and often prevents the patient from sleeping on the affected side. Many treatments have been described in the literature, but the condition is prone to recurrence. A literature search was performed in order to identify the best possible treatment. Fifty-eight articles were included, describing and investigating nonsurgical as well as surgical treatment modalities. Large prospective, controlled, and randomised long-term studies are lacking, but based on the available literature, we recommend starting with a conservative approach using decompression devices. Simple surgical procedures should only be used if conservative measures fail.

## 1. Introduction

Chondrodermatitis Nodularis Helicis (CNH) is an inflammatory process affecting the skin and cartilage of the ear. Typically, it presents as a painful nodule of the helix and to a lesser extent the antihelix of the ear. Therefore, some have suggested changing the name to chondrodermatitis nodularis auricularis, as lesions are seen both on the helix and on the antihelix [1] and have even been reported on the posterior ear and in the external auditory canal [2, 3].

The condition was first described by the dermatologist Winkler in 1915 [4], who reported eight cases and soon afterwards by Foerster, who reported a further four cases [5]. Foerster later defined the condition in 1925 [6], with an additional eight cases, describing the clinical and microscopic features and the treatment of the condition.

The anatomy of the ear is unique, because the skin of the anterior and posterior surface is adherent to the perichondrium and is devoid of subcutaneous fat. The blood supply is provided by a rich subdermal plexus of vessels lying in the fascial layer between the skin and the perichondrium. The arterial supply to the auricle is derived from branches of the superficial temporal artery and the postauricular artery, so that the lateral surface of the ear has a dual arterial supply [7].

## 2. Method and Material

This paper is presented as a literature review. A PubMed search was performed on the 18th of December 2015 using the search term “chondrodermatitis”, identifying 134 articles. Primarily, abstracts of articles from 1996 onwards were reviewed leading to a full article review according to relevance. Articles with special focus on treatment modalities were included. Reference lists of selected articles were reviewed for other relevant articles.

## 3. Results

Fifty-eight articles were included: 13 prospective studies [8–20], 16 retrospective studies [1, 21–35], 4 case series [36–39], 13 case reports [2, 3, 40–50], 8 descriptions of treatment methods [7, 51–57], and 4 reviews [58–61].

In general, the literature on CNH is of fairly low quality, as it includes many case reports and retrospective studies. The 13 prospective studies are neither randomised, blinded nor controlled, although one histopathological study, investigating nerve hyperplasia, did include other tumours of the ear as controls. Most of the prospective studies involve only small cohorts of patients ranging from 5 to 99. Many articles lack information on anamnesis, symptoms, clinical signs, or length of follow-up as well as a clear definition of outcome (i.e., is a successful outcome reduced pain, absence of pain, reduction in size of nodule, or complete resolution of the nodule?). The follow-up period is generally short, ranging from 2 to 12 months, although a few studies report on follow-up intervals of up to 41–96 months. Another weakness in most of the studies is the presence of confounding treatment modalities: many patients had previously tried other treatments, some were subjected to more than one treatment, and many were advised to decompress the lesion in conjunction with the investigated treatment.

### 3.1. Clinical Presentation

**Figure 1:**
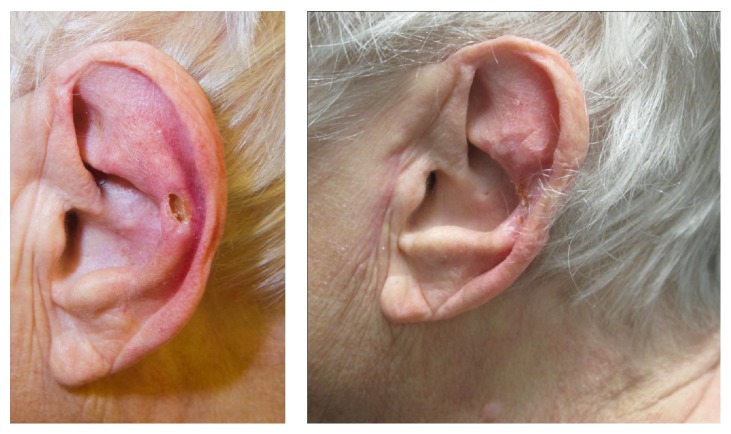
Pre- and postoperative pictures of a patient with CNH. 79-year-old woman with recurrence of CNH on the left antihelix. Two years prior to referral the patient was treated surgically (unknown method) for CNH in the same location. Recurrence occurred 3 months before referral and was initially treated with topical sodium fusidate with no effect. Treated with excision of skin and underlying cartilage and coverage of the defect with full thickness skin graft, all symptoms resolved and an acceptable cosmetic outcome was obtained at 3-month follow-up.

CNH typically presents with an oval shaped nodule with elevated edges and a central crust or depression, usually 4–6 mm in diameter and frequently surrounded by an area of erythema. The nodule is usually firm to touch and bound to the underlying cartilage. It is characterised by exquisite tenderness, which often prevents the patient from sleeping on the affected side. Typically, the nodule is located on the outer ear (for men often on the helix and for women often on the antihelix) but locations on the posterior ear [2] and in the external auditory canal [3] have also been reported (see Figure 1). The lesions are typically located on the ear of the preferred sleeping-side, the right side being predominant, and are typically unilateral, although bilateral lesions have also been reported [8, 40–42]. Nocturnal pain is by far the most common symptom, with only few complaints of pain during the day, except when touched. Other features associated with the nodules include crusting, bleeding, and exudate, which can make it difficult to exclude skin malignancy.

### 3.2. Gender and Age Distribution

Historically, the literature describes a striking preponderance of males (10 : 1). In this review, 27 studies reported on the gender distribution, including a total of 628 males and 452 females, resulting in a male to female ratio of 1 : 0.7. As the exact cause of CNH is unknown, any discussion on the reasons for the gender difference is speculative.

CNH most commonly presents in patients over 40, although there have been isolated reports of children also affected [43–45]. Excluding all case reports and including only studies where a mean age is reported, 21 studies reported a mean age from 43 to 76 years.

Studies on incidence and prevalence were not identified, but anecdotally, our pathology department reports that the incidence is common.

### 3.3. Pathogenesis

The pathomechanism of CNH has not yet been fully uncovered. The striking and repeated observation that CNH is usually unilateral and usually affects the ear of the preferred sleeping-side suggests that pressure damage, predominantly from the weight of the head during sleep, is the most important aetiological factor. CNH arising from pressure due to hearing aids and other headgear has also been described [9, 21, 42, 44, 46]. Other aetiological factors such as trauma from cold (frostbite) and actinic damage have also been suggested [9, 21–23, 40, 42–47, 58].

It is possible that anatomical features of the individual (e.g., a grossly protruding helix or antihelix) predispose to the condition, but in most cases, the cartilage does not have to be grossly abnormal to initiate the disease. As the ear-shape does not change much during adult life, other factors must cause development of the disease in an otherwise unaffected ear. This could be due to distortion or calcification due to injury or simply because the cartilage becomes less flexible with increasing age and hence more vulnerable to pressure damage. It is also possible that the difference in site distribution between men and women is explained by the presence of a more protuberant helix in men and a more protuberant antihelix in women [24].

**Figure 2:**
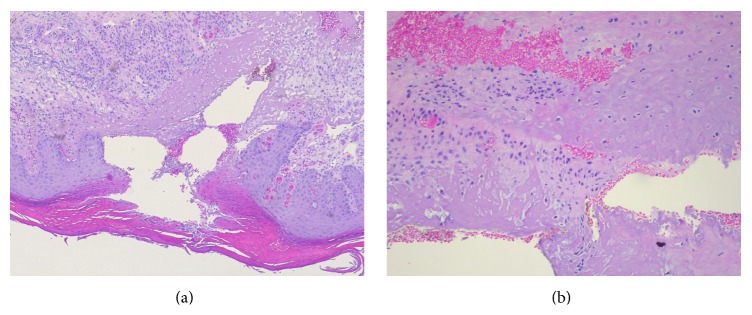
Classical histopathology of CNH. (a) shows the classical findings of hyperkeratosis, parakeratosis, adjacent hyperplasia of the epithelium, and substantial destruction of the dermal tissue lined by sclerosis and proliferation of small vessels. (b) shows the destruction of cartilage.

It has been suggested that pressure or repeated trauma may lead to ischemia of the cartilage and the auricular perichondrium. This arises because of the lack of protection of a thick subcutaneous tissue and changes in the perichondrial arterioles. Although dated, this vasculitis/inflammatory theory, first proposed by Halter in 1936 [62], is still the most widely accepted explanation for CNH. More recently, it was supported by Upile et al. in 2009 [23], with a histopathological review of 16 cases. Upile et al. confirmed the previous findings of epidermal acanthosis associated with a horny, partly parakeratotic, plug or ulceration and scale crust, superficial debris with fibrin, sclerosis, perichondrial fibrosis, and a varying degree of cartilage degeneration closely associated with areas of granulation tissue (see Figure 2). Upile et al. also confirmed the presence of consistent arteriolar narrowing in the part of the perichondrium most remote from the arterial blood supply, that is, the helix. This leads to ischemic changes and death of the metabolically active underlying cartilage with necrosis and extrusion and to severe local inflammation secondary to a foreign body reaction [23]. Furthermore, nerve hyperplasia is present in CNH, though often masked by the intense vascular and inflammatory reactions, which may explain the characteristic exquisite tenderness [10].

A possible association with systemic disease, such as dermatomyositis and systemic sclerosis, has also been suggested [11]. However, it is more likely that immobility caused by these diseases, and hence the inability to alter sleeping position, could be the true cause of a correlation between CNH and systemic disease.

### 3.4. Differential Diagnosis

The differential diagnosis depends on the clinical presentation, but the location of the lesion in combination with exquisite tenderness helps to differentiate CNH from other diagnoses. Gouty tophi may be suspected, when there are multiple lesions, not only on the ear, but also on fingers and toes. The nodular appearance with a central ulceration can mimic a basal cell carcinoma, while larger and more inflamed lesions may look like a squamous cell carcinoma, one of the most common misdiagnoses of CNH. Keratoacanthoma usually exhibits much faster growth, resulting in a larger tumour volume and with the classic resolution over months. When keratosis is the predominant clinical feature, an actinic or seborrheic keratosis may be suspected. However, only histopathological examination of a deep biopsy can secure the diagnosis and definitely rule out malignancy.

### 3.5. Treatment

In general, the scientific work on the treatment of CNH lacks large, randomised, controlled studies. Historically, surgical excision (Table 3) has been more frequently investigated and the preferred treatment modality [1, 8, 22, 24, 26, 32–35, 39, 50]. Recently, however, more conservative methods have been investigated in an effort to avoid the discomfort of surgery, the risk of postoperative infections, and problems with wound healing and deformity (Table 1).

#### 3.5.1. Pressure Relieving Padding

The repeated observation that CNH usually affects the ear on the preferred sleeping-side suggests that pressure on the ear is an important aetiological factor. This leads to the logical conclusion that relieving pressure on the ear will be a successful treatment. Seven studies, including 148 patients treated with pressure-relieving padding alone, were identified (Table 1). Many of the studies on other treatment modalities also recommend decompression of the ear after treatment, making this a major confounding factor. Half of the studies on pressure relieving padding were prospective, but none were randomized or controlled. Several methods were described, including self-adhering foam behind the ear, foam bandages strapped to the head, and sleeping on a doughnut-shaped pillow. All aimed to provide a means for relieving the pressure on the affected area of the ear. The cure rate reported in the pure decompression studies was as low as 57% and as high as 92%. In 2011 Durrant et al. published a prospective study on 75 patients treated with a customised ear prosthesis [12] where 47% experienced complete resolution and 27% improvement. After more than 6 months, 31% experienced recurrence, but overall 91% of the patients avoided surgery [12]. Considering all the studies on pressure relieving padding, we conclude that this conservative treatment-approach is inexpensive and cost-effective but that the result depends very much on the compliance of the patient.

#### 3.5.2. Topical Nitroglycerin

As CNH may result from chondrial ischemia arising from perichondrial arteriolar narrowing and nitroglycerin causes relaxation and vasodilation of the arteriolar smooth muscle, this treatment may restore adequate blood flow to reverse the ischemic changes [15]. Four studies totalling 53 patients treated with topical nitroglycerin were identified (Table 1). Forty-two patients were treated with nitroglycerin gel applied once or twice daily for up to three months and 11 patients were treated with a transdermal nitroglycerin patch, 12 hours a day for two months. Of the latter, two patients stopped the treatment due to moderate headaches and one patient only had a partial response and later underwent surgery, leaving 64% who had a complete response. Of the former, a 93% and 92% cure rate was reported. Topical nitroglycerin is usually used in the treatment of angina pectoris or chronic anal fissures and the most common side effect is transient headaches. Skin irritation has also been reported. Due to the low systemic absorption from the ear, topical treatment should not cause any major side effects [15]. We conclude that this treatment modality shows promising results, but further studies, with larger numbers of patients, are needed.

#### 3.5.3. Glucocorticoid Injection

As explained above, the aetiology of CNH probably includes local inflammation secondary to a foreign body reaction. Glucocorticoids are potent anti-inflammatory agents and should be able to stop the local inflammation. Three studies on intralesional injection of glucocorticoid were identified, one prospective (Table 1). A study by Lawrence from 1991 was actually a study of the effectiveness of surgery, but 44 patients first received intralesional steroid injections and those who had not responded after 8 weeks (73%) were then offered surgery [8]. Also noteworthy is the study by Cox and Denham from 2002 [29], who retrospectively reported the outcomes of 60 patients treated with one dose of 0,1 mL of intralesional triamcinolone, demonstrating a 40% response at three months. Four patients later required a second injection and were then symptom-free. Another four patients had late recurrence, decreasing the long-term success rate to 33%, after follow-up of up to eight years [29]. Although this treatment is easy, fast, and inexpensive, we do not feel it should be recommended as first line treatment, due to the low cure rates.

#### 3.5.4. Injectable Collagen

Greenbaum reported on the outcome of treatment for 5 patients in 1991, who received injections of collagen over the perichondrium to relieve pressure on the cartilage (Table 1). The cure rate was reported to be 100% after a follow-up period of 0–16 months [17]. However, such results are inconclusive due to the small number of patients and relative short period of follow-up.

#### 3.5.5. PDT

It is thought that PDT acts on several pathways involved in CNH: it has an anti-inflammatory and immunomodulatory action, an effect on vascularisation and on collagen and it may also have a chondroprotective effect [30]. Treatment with aminolevulinic acid- (ALA-) PDT seems to slow the inflammatory reaction in the skin, because of the death of the resident macrophages and mast cells and the slow recovery to cytokine responsiveness of the surviving cell population. Although it is generally accepted that PDT causes acute inflammation, it can also interrupt the process of chronic inflammation and stimulate healing [30]. It has been shown that blood perfusion is increased immediately after irradiation and this persists for up to one week. It has also been shown that chondrocytes are not destroyed, but that PDT modulates in vitro cartilage metabolism, activating a chondroprotective effect in photosensitized cartilage in the context of osteoarthritis [30]. Two studies, involving only seven patients treated with PDT, were identified (Table 2). Gilaberte et al. reported on five patients treated with PDT, after preparing the site with curettage to scrape away crusts, and so forth. The cure rate was reported to be 80%, although the period of follow-up was not reported [30]. These results should be considered inconclusive because of the very small numbers of patients treated and the limited follow-up.

#### 3.5.6. CO_2_ and Argon Laser

Only two studies, comprising 14 patients, were identified [19, 38] (Table 2). Patients received treatment with a CO_2_ laser and their wounds were allowed to heal by secondary intention. However, they were also instructed to decompress their ears for 3-4 weeks. The cure rate was reported as 100%, after a follow-up period of 2–24 months. One study reported on the outcomes of nine patients treated with argon laser, with a 56% cure rate [18]. The small number of patients in these studies makes it difficult to form any definite conclusion, though the high cure rates warrant further studies.

#### 3.5.7. Curettage/Electrocauterization

Kromann et al. published the most comprehensive study of a nonsurgical treatment for CNH in 1983 [21] (Table 2). They reported the outcomes of 142 cases during a 15-year period who were treated with curettage followed by electrocauterization. Fifteen patients also underwent treatment with radiotherapy after curettage. A further five patients were treated with radiotherapy alone, with no effect, and then proceeded to curettage with electrocauterization. An unknown number received treatment with intralesional triamcinolone injections and freezing with carbon dioxide in addition to curettage. Seventy-eight patients were reexamined after an average of 7.1 years and 31% were found to have relapsed. An overall recurrence rate of 25% was reported, but after an unknown follow-up [21]. Although curettage appears to have an acceptable cure and recurrence rate, there are many confounding variables, making it difficult to know with certainty whether curettage alone was responsible for the “cures” achieved. Moreover, there have been no other studies of this treatment since 1983.

#### 3.5.8. Surgery

Many techniques have been described and most agree that a simple approach, with primary excision and meticulous trimming of the cartilage, is the preferred method [8, 11, 22, 24, 26, 32–35, 39, 43, 50].

Seventeen surgical studies, including more than 500 patients, were reviewed (Table 3). The surgical techniques described include wide excision with reconstruction of the ear using local flaps, skin grafts, excision of the affected skin and underlying cartilage, and skin-sparing techniques with excision of the cartilage only.

The cure rates reported range from 66% to 100% with a recurrence rate of up to 38%. In this review, recurrences were most frequent at the edges of the cartilage defect. Therefore, later studies advised greater attention to careful trimming of all affected cartilage.

In 1991, Lawrence [8] described a surgical technique to remove only the affected cartilage under a skin flap. With a cure rate of 88% and a recurrence rate of 12% this technique proved to be as efficient as any other at the time and has since been described in various modifications in an effort to simplify the procedure [8].

In 2014, Kulendra et al. described a method used to treat 59 patients [31]. On the helix, a skin incision was made which was 2-3 times the diameter of the nodule, 1-2 mm wide, and tapered to a point. The nodule and adjacent area were excised and sent for histology. The exposed, raised cartilage was gradually removed at the centre, using a surgical blade, sharp-pointed-scissors, or a diamond burr. The wound was then closed directly with interrupted sutures. On the antihelix, access to the nodule was achieved through an anterior interhelical and inferior releasing incision, elevating a skin flap with perichondrium to expose the cartilage. The nodule and surrounding cartilage were excised, avoiding sharp edges and removing any of the remaining antihelix to a height lower than the helix. The skin flap was then scarified and sutured back in place. These techniques resulted in a cure rate of 88% for the helix and 89% for the antihelix after a follow-up of 85 months [31].

Some authors stress the importance of sending material, including skin, for histology to confirm the diagnosis and rule out malignancy. Rajan and Langtry, 2007 [32], support this argument and recommend their “punch and graft technique” used on 22 patients with a cure rate of 83% after a follow-up of 1–86 months. A punch biopsy instrument, with a diameter large enough to encompass the lesion, was used to cut the skin and the underlying cartilage. The specimen was then excised with scissors or blade and sent for histology. The same punch tool was used to harvest a full thickness skin graft from the postauricular area and the graft fixed in place with 6-0 interrupted sutures [32].

## 4. Conclusion

Even though CNH is not life threatening, it can impair quality of life. The incidence and prevalence is not known, but the disease appears to be common. Historically, a preponderance for males has been reported but in this review, we found a more equal gender distribution.

The vasculitis/inflammatory theory is most widely supported and explains the development of the lesion by arteriolar narrowing in the perichondrium leading to ischemic changes in the cartilage, followed by necrosis and extrusion of the necrotic material. This theory is further supported by the effectiveness of therapies aimed at improvement of the blood supply and/or dilation of blood vessels. One such therapy is topical nitroglycerin, which shows promising results, but larger studies are still warranted.

Much attention has recently been paid to decompression therapy and this seems to be a cost-effective therapy with acceptable cure rates but obviously depends on the compliance of the patient.

Multiple other therapies have been investigated, but in order to establish guidelines for the best treatment of CNH, larger, prospective, controlled, and randomised long-term studies are still required.

Based on the available literature however, we suggest first-line treatment with decompression devices and only if these are not effective surgical treatment. The surgical method recommended should be with minimal skin excision, primarily for histology, excision of the affected cartilage with careful trimming of the cartilage edges, and when possible direct skin closure, otherwise a skin graft.

## Figures and Tables

**Table 1:** Topical treatment.

First author and year	Number of patients	Method	Cure rate	Follow-up (mean)	Recurrence rate	Pros and cons
*Pressure relieving padding*						
Travelute 2013 [25]	10	Self-adhering foam	90%	Not reported	0	Retrospective. Follow-up not reported.
Naqash 2013 [59]	1	Ear-padding	Pain resolved	6 weeks	0	Case report. Short follow-up.
Kuen-Spiegl 2011 [9]	12 (18)	Bandage + foam plastic	92%	41–61 months	25% (but discontinued treatment)	Prospective. 6 did not comply.
Durrant 2011 [12]	75	Customized ear prosthesis	74%	52 months	31%	Prospective. Confounder with topical steroid.
Sanu 2007 [13]	23	Doughnut pillow	57%	12 months	0	Prospective. 7 did not comply.
Moncrieff 2004 [26]	15	Foam-padding strapped to head	87%	18 weeks	0	Retrospective. Short follow-up.
Timoney 2002 [14]	12	Different decompression methods	92%	24 months	8%	Prospective. Used several methods.

*Topical nitroglycerin*						
Sanz-Motilva 2015 [27]	29	0,2% topical nitroglycerin	93%	5,9 months	0,03%	Retrospective. Confounder with decompression.
Colmenero 2014 [15]	11	Transdermal nitroglycerin patch	64%	3 months	0	Prospective. Lack of histology.
Yélamos 2013 [48]	1	2% nitroglycerin gel	Good effect	4 months	0	Case report.
Flynn 2011 [28]	12 pt/13 lesions	2% topical nitroglycerin	92%	2–13 months	0 (within follow-up period)	Retrospective. Wide range of follow-up.

*Glucocorticoid injection*						
Cox 2002 [29]	60	1x triamcinolone intralesionally	40% 3 months/33% long-term	3–96 months	6%	Retrospective.
Lawrence 1991 [8]	44	Triamcinolone, 0,2–0,5 mL intralesionally + topical betamethasone ×2 daily + lidocaine gel 2% before bed for 8 weeks	27% at 8 weeks	16 months (4,5–34)	Non-respondents went on to surgery. Respondents had 0 recurrence after 14 months	Prospective. Confounding therapies.
Schmidt 1984 [36]	3	Local infiltration with Prednisolon-crystal-suspension, 3–6 inj.	67%	1 week after last treatment	Not reported	Case series. No follow-up after last treatment.
Wade 1979 [16]	8	Intralesional inj. with triamcinolone	100%	0–12 months	0	Prospective. Unsure follow-up.

*Injectable collagen*						
Greenbaum 1991 [17]	5	Collagen inj. over perichondrium	100%	16 months	0	Prospective.

**Table 2:** Physical therapies.

First author and year	Number of patients	Method	Cure rate	Follow-up (mean)	Recurrence rate	Pros and cons
*Photodynamic therapy (PDT)*						
Pellegrino 2011 [37]	2	PDT	100%	9 months	0	Case report.
Gilaberte 2010 [30]	5	PDT + curretage	80%	Not reported	Not reported	Retrospective, confounder with curettage, follow-up not reported.

*Cryotherapy*						
Senel 2010 [47]	1	Cryotherapy ×2	100%	12 months	0	Case report.

*Argon laser*						
Hesse 1994 [18]	9 pts/16 lesions	Biopsy + argon laser for surface and underlying cartilage	56%	3–16 months	“No real recurrence” (4 patients with recurrence after 3–16 months, retreated)	Prospective.

*CO*_*2*_ * laser*						
Taylor 1991 [19]	11 pts/12 lesions	CO_2_ laser to vaporize the cutaneous nodule and involved cartilage + decompression for 3-4 weeks	100%	2–15 months	0	Prospective. Confounder with decompression.
Karam 1988 [38]	3	CO_2_ laser at 15 W	100%	24 months	0	Case series.

*Curettage/electrocauterization*						
Kromann 1983 [21]	142	Curettage followed by electrocauterization	69%	7,1 years (average)	31%	Retrospective. Confounder with many other therapies.

**Table 3:** Surgical treatment.

First author and year	Method	Patients	Follow-up	Cure rate (%)	Recurrence rate	Pros and cons
Kulendra 2014 [31]	Excision of nodule + rim shave + direct suture + decompression	59 pt/65 lesions	85 months	88% for the helix and 89% for the antihelix	12% for the helix, 11 for the antihelix	Retrospective. Confounder with decompression.

Magliulo 2014 [3]	Excision + skin graft	1	12 months	100%	No recurrence	Case report. No history, no picture.

Feldman 2009 [1]	Wedge resection/circumferential excision of skin + underlying cartilage + skin graft	55 pt/62 lesions	Not reported	89%/96%	11% (infection) only 30% of these needed reexcision	Retrospective. No report of follow-up.

Hussain 2009 [22]	No skin excision, only nodule + trimming of cartilage	34	4 months	94%	0 (6% did not respond)	Retrospective.

Chan 2009 [49]	Surgically excised	2	12 months	100%	0	Case report.

Dreiman 2007 [7]	Wedge-incision w Antia Buch reconstruction.	1	8 months	100%	0	Case report.

Rajan 2007 [32]	Punch and graft technique	22 pt/23 lesions	1–86 months	83%	18%	Retrospective.

Tsung-Hua 2007 [43]	Excision of nodule + decompression	1	6 months	100%	0	Case report. Confounder with decompression.

Grigoryants 2007 [44]	Exc. of skin + cartilage + skin graft + decompression	1	6 months	100%	0	Case report. Confounding treatment modalities.

Rex 2006 [33]	Narrow elliptical skin excision + cartilage shaving	74	Helix: 54 (14–90) months Antihelix: 50 (11–78) months	Helix: 90% Antihelix: 63%	Helix: 11% Antihelix: 38%	Retrospective.

Jacob K 2005 [50]	Excision of cartilage (+ necrotic muscle)	1	6 months	100%	0	Case report surgical procedure not well described.

Moncrieff 2004 [26]	Excision biopsy of nodule, paring the underlying cartilage, foam-padding	41	77 (15–150) weeks	66%	34%	Retrospective review, confounding treatment modalities.

De Ru 2002 [34]	Skin-sparing cartilage resection. All previously treated with Kenacort inj.	34 pt/37 lesions	31 months (3–111)	92%	3%	Retrospective. Confounding treatment modalities.

Hudson-Peacock 1999 [24]	Punch biopsy + cartilage resection + shaving + suture of skin	77	52 (helix) + 55 (antihelix) months	84% (helix) 75% (antihelix)	Helix: 16% Antihelix: 25% (all in excision margin)	Retrospective.

Zuber 1999 [39]	Modified shave-technique followed by electrosurgical feathering	4	Not reported	Not reported	Not reported	No relevant information available.

Munnoch 1996 [35]	Minimal skin excision and extensive cartilage resection	50	37 months (18–78)	100%	0	Retrospective.

Lawrence 1991 [8]	Intralesional steroid therapy/excision of cartilage without skin excision	46	16 months	88%	12% (at excision margins)	Confounding treatment modalities.

## References

[1] Feldman A. L., Manstein C. H., Manstein M. E., Czulewicz A. (2009). Chondrodermatitis nodularis auricularis: a new name for an old disease. *Plastic and Reconstructive Surgery*.

[2] Cox N. H. (2002). Posterior auricular chondrodermatitis nodularis. *Clinical & Experimental Dermatology*.

[3] Magliulo G., Iannella G., Moretti V., Re M. (2014). Chondrodermatitis nodularis chronica and external auditory canal. *Otology and Neurotology*.

[4] Winkler M. (1915). Knötcehnformige Erkrankung am helix. Chondrodermatitis nodularis chronic helicis. *Archiv für Dermatologie und Syphilis*.

[5] Foerster O. H. (1918). A painful nodular growth of the ear. *The Journal of Cutaneous Diseases*.

[6] Foerster O. H. (1925). Painful nodular growth of the ear. *Archives of Dermatology and Syphilology*.

[7] Dreiman B. B. (2007). Chondrodermatitis nodularis chronica helicis treated with antia-buch reconstruction: review and case report. *Journal of Oral and Maxillofacial Surgery*.

[8] Lawrence C. M. (1991). The treatment of chondrodermatitis nodularis with cartilage removal alone. *Archives of Dermatology*.

[9] Kuen-Spiegl M., Ratzinger G., Sepp N., Fritsch P. (2011). Chondrodermatitis nodularis chronica helicis—a conservative therapeutic approach by decompression. *Journal der Deutschen Dermatologischen Gesellschaft*.

[10] Cribier B., Scrivener Y., Peltre B. (2006). Neural hyperplasia in chondrodermatitis nodularis chronica helicis. *Journal of the American Academy of Dermatology*.

[11] Magro C. M., Frambach G. E., Crowson A. N. (2005). Chondrodermatitis nodularis helicis as a marker of internal syndromes associated with microvascular injury. *Journal of Cutaneous Pathology*.

[12] Durrant C. A. T., Lloyd-Hughes H., Worth R., Allen D. L., Pereira J. (2011). Auricular pressure-relieving cushions for treatment of chondrodermatitis nodularis helicis: a series of 75 cases and a review of the literature. *European Journal of Plastic Surgery*.

[13] Sanu A., Koppana R., Snow D. G. (2007). Management of chondrodermatitis nodularis chronica helicis using a ‘doughnut pillow’. *Journal of Laryngology and Otology*.

[14] Timoney N., Davison P. M. (2002). Management of chondrodermatitis helicis by protective padding: a series of 12 cases and a review of the literature. *British Journal of Plastic Surgery*.

[15] Colmenero C. G., Garćia E. M., Morente G. B., Sánchez J. T. (2014). Nitroglycerin patch for the treatment of chondrodermatitis nodularis helicis: a new therapeutic option. *Dermatologic Therapy*.

[16] Wade T. R. (1979). Chondrodermatitis nodularis chronica helicis. A review with emphasis on steroid therapy. *Cutis*.

[17] Greenbaum S. S. (1991). The treatment of chondrodermatitis nodularis chronica helicis with injectable collagen. *International Journal of Dermatology*.

[18] Hesse G., Schmoeckel C., Wichmann-Hesse A. (1994). Argon laser therapy for chondrodermatitis nodularis chronica helicis. *Hautarzt*.

[19] Taylor M. B. (1991). Chondrodermatitis nodularis chronica helicis: successful treatment with the carbon dioxide laser. *Journal of Dermatologic Surgery and Oncology*.

[20] Wettlé C., Keller F., Will F., Lefebvre F., Cribier B. (2013). Chondrodermatitis nodularis chronica helicis: a descriptive study of 99 patients. *Annales de Dermatologie et de Venereologie*.

[21] Kromann N., Høyer H., Reymann F. (1983). Chondrodermatitis nodularis chronica helicis treated with curettage and electrocauterization: follow-up of a 15-year material. *Acta Dermato-Venereologica*.

[22] Hussain W., Chalmers R. J. G. (2009). Simplified surgical treatment of chondrodermatitis nodularis by cartilage trimming and sutureless skin closure. *British Journal of Dermatology*.

[23] Upile T., Patel N. N., Jerjes W., Singh N. U., Sandison A., Michaels L. (2009). Advances in the understanding of chondrodermatitis nodularis chronica helices: the perichondrial vasculitis theory. *Clinical Otolaryngology*.

[24] Hudson-Peacock M. J., Cox N. H., Lawrence C. M. (1999). The long-term results of cartilage removal alone for the treatment of chondrodermatitis nodularis. *British Journal of Dermatology*.

[25] Travelute C. R. (2013). Self-adhering foam: a simple method for pressure relief during sleep in patients with chondrodermatitis nodularis helicis. *Dermatologic Surgery*.

[26] Moncrieff M., Sassoon E. M. (2004). Effective treatment of chondrodermatitis nodularis chronica helicis using a conservative approach. *British Journal of Dermatology*.

[27] Sanz-Motilva V., Martorell-Calatayud A., García-Rodrigo C. G. (2015). The usefulness of 0.2% topical nitroglycerin for chondrodermatitis nodularis helicis. *Actas Dermo-Sifiliográficas*.

[28] Flynn V., Chisholm C., Grimwood R. (2011). Topical nitroglycerin: a promising treatment option for chondrodermatitis nodularis helicis. *Journal of the American Academy of Dermatology*.

[29] Cox N. H., Denham P. F. (2002). Intralesional triamcinolone for chondrodermatitis nodularis: a follow-up study of 60 patients. *British Journal of Dermatology*.

[30] Gilaberte Y., Frias M. P., Pérez-Lorenz J. B. (2010). Chondrodermatitis nodularis helicis successfully treated with photodynamic therapy. *Archives of Dermatology*.

[31] Kulendra K., Upile T., Salim F., O'Connor T., Hasnie A., Phillips D. E. (2014). Long-term recurrence rates following excision and cartilage rim shave of chondrodermatitis nodularis chronica helicis and antihelicis. *Clinical Otolaryngology*.

[32] Rajan N., Langtry J. A. A. (2007). The punch and graft technique: a novel method of surgical treatment for chondrodermatitis nodularis helicis. *British Journal of Dermatology*.

[33] Rex J., Ribera M., Bielsa I., Mangas C., Xifra A., Ferrándiz C. (2006). Narrow elliptical skin excision and cartilage shaving for treatment of chondrodermatitis nodularis. *Dermatologic Surgery*.

[34] De Ru J. A., Lohuis P. J. F. M., Saleh H. A., Vuyk H. D. (2002). Treatment of chondrodermatitis nodularis with removal of the underlying cartilage alone: retrospective analysis of experience in 37 lesions. *Journal of Laryngology and Otology*.

[35] Munnoch D. A., Herbert K. J., Morris A. M. (1996). Chondrodermatitis nodularis chronica helicis et antihelicis. *British Journal of Plastic Surgery*.

[36] Schmidt C., Heise H., Flegel H. (1984). Treatment of chronic nodular chondrodermatitis helicis with a suspension of prednisolone crystals. *Dermatologische Monatsschrift*.

[37] Pellegrino M., Taddeucci P., Mei S., Peccianti C., Fimiani M. (2011). Chondrodermatitis nodularis chronica helicis and photodynamic therapy: a new therapeutic option?. *Dermatologic Therapy*.

[38] Karam F., Bauman T. (1988). Carbon dioxide laser treatment for chondrodermatitis nodularis chronica helicis. *Ear, Nose and Throat Journal*.

[39] Zuber T. J., Jackson E. (1999). Chondrodermatitis nodularis chronica helicis. *Archives of Family Medicine*.

[40] Kaur R. R., Lee A. D., Feldman S. R. (2010). Bilateral chondrodermatitis nodularis chronica helicis on the antihelix in an elderly woman. *International Journal of Dermatology*.

[41] Oelzner S., Elsner P. (2003). Bilateral chondrodermatitis nodularis chronica helicis on the free border of the helix in a woman. *Journal of the American Academy of Dermatology*.

[42] Khurana U., Solanki L. S., Dhingra M. (2015). A man with painful nodules on both ears. *JAMA Otolaryngology-Head & Neck Surgery*.

[43] Tsung-Hua T., Yang-Chih L., Hsiu-Chin C. (2007). Infantile chondrodermatitis nodularis. *Pediatric Dermatology*.

[44] Grigoryants V., Qureshi H., Patterson J. W., Lin K. Y. (2007). Pediatric chondrodermatitis nodularis helicis. *Journal of Craniofacial Surgery*.

[45] Rogers N. E., Farris P. K., Wang A. R. (2003). Juvenile Chondrodermatitis Nodularis Helicis: a case report and literature review. *Pediatric Dermatology*.

[46] Ortiz A., Martín P., Domínguez J., Conejo-Mir J. (2015). Cell phone-induced chondrodermatitis nodularis antihelicis. *Actas Dermo-Sifiliográficas*.

[47] Senel E. (2010). Chondrodermatitis nodularis chronica helicis. *Clinical Medicine Insights: Dermatology*.

[48] Yélamos O., Dalmau J., Puig L. (2013). Chondrodermatitis nodularis helicis: successful treatment with 2% nitroglycerin gel. *Actas Dermo-Sifiliograficas*.

[49] Chan H. P., Neuhaus I. M., Maibach H. I. (2009). Chondrodermatitis nodularis chronica helicis in monozygotic twins. *Clinical and Experimental Dermatology*.

[50] Jacob K J., Satheesh S., Menon P., Saju K. G. (2005). Winkler's disease. *Indian Journal of Otolaryngology and Head and Neck Surgery*.

[51] Jain P. K., Jain S. (2005). Use of disposable curette in the treatment of Chondrodermatitis Nodularis Helicis. *Clinical Otolaryngology*.

[52] Ormond P., Collins P. (2004). Modified surgical excision for the treatment of chondrodermatitis nodularis. *Dermatologic Surgery*.

[53] Long D., Maloney M. E. (1996). Surgical pearl: surgical planing in the treatment of chondrodermatitis nodularis chronica helicis of the antihelix. *Journal of the American Academy of Dermatology*.

[54] Coldiron B. M. (1991). The surgical management of chondrodermatitis nodularis chronica helicis. *Journal of Dermatologic Surgery and Oncology*.

[55] Bruns N. M., Hessam S., Valavanis K., Scholl L., Bechara F. G. (2015). Surgical treatment of chondrodermatitis nodularis helicis via a retroauricular incision. *Journal der Deutschen Dermatologischen Gesellschaft*.

[56] Yaneza M. M. C., Sheikh S. (2013). Chondrodermatitis nodularis chronica helicis excision and reconstruction. *Journal of Laryngology and Otology*.

[57] Cognetta A. B., Wolfe C. M., Green W. H., Hatfield H. K. (2012). Triangular window technique: a novel approach for the surgical treatment of chondrodermatitis nodularis helicis. *Dermatologic Surgery*.

[58] Sehgal V. N., Singh N. (2009). Chondrodermatitis nodularis. *The American Journal of Otolaryngology—Head and Neck Medicine and Surgery*.

[59] Naqash M. M., Salati S. A. (2013). Chondrodermatitis nodularis chronica helicis—a review. *Journal of Pakistan Association of Dermatologists*.

[60] Wagner G., Liefeith J., Sachse M. M. (2011). Clinical appearance, differential diagnoses and therapeutical options of chondrodermatitis nodularis chronica helicis Winkler. *Journal of the German Society of Dermatology*.

[61] Singh M., Wilson A., Parkinson S. (2009). Two non-surgical treatments for chondrodermatitis nodularis helicis. *British Journal of Oral and Maxillofacial Surgery*.

[62] Halter K. (1936). Zur Pathogenese der Chondrodermatitis nodularis chron. helicis. *Dermatology*.

